# Family-Centered Care at Pediatric Cardiac Intensive Care Units in Germany and the Relationship With Parent and Infant Well-Being: A Study Protocol

**DOI:** 10.3389/fped.2021.666904

**Published:** 2021-08-12

**Authors:** Hannah Ferentzi, Ralph C. A. Rippe, Jos M. Latour, Stephan Schubert, Alona Girch, Michaela Jönebratt Stocker, Constanze Pfitzer, Joachim Photiadis, Eugen Sandica, Felix Berger, Katharina R. L. Schmitt

**Affiliations:** ^1^Department of Pediatric Cardiology, German Heart Center Berlin, Berlin, Germany; ^2^Research Methods and Statistics, Institute of Education and Child Studies, Leiden University, Leiden, Netherlands; ^3^School of Nursing and Midwifery, Faculty of Health, University of Plymouth, Plymouth, United Kingdom; ^4^Center for Congenital Heart Disease, Heart- and Diabetescenter NRW, University Clinic of Ruhr-University Bochum (RUB), Bad Oeynhausen, Germany; ^5^Department of Pediatric Cardiology, Charité University Hospital Berlin, Berlin, Germany; ^6^Berlin Institute of Health, Charité University Hospital Berlin, Berlin, Germany

**Keywords:** intensive care unit, parental mental well-being, infant physical well-being, children, congenital heart disease, infant mental well-being, family-centered care, pediatric cardiology

## Abstract

**Rationale and Aim:** Infants with Congenital Heart Disease (CHD) are at risk for neurodevelopmental delays, emotional, social and behavioral difficulties. Hospitalization early in life and associated stressors may contribute to these challenges. Family-centered Care (FCC) is a health care approach that is respectful of and responsive to the needs and values of a family and has shown to be effective in improving health outcomes of premature infants, as well as the mental well-being of their parents. However, there is limited empirical data available on FCC practices in pediatric cardiology and associations with parent and infant outcomes.

**Methods and Analysis:** In this cross-sectional study, we will explore FCC practices at two pediatric cardiac intensive care units in Germany, assess parent satisfaction with FCC, and investigate associations with parental mental well-being and parenting stress, as well as infant physical and mental well-being. We will collect data of 280 infants with CHD and their families. Data will be analyzed using multivariate statistics and multilevel modeling.

**Implications and Dissemination:** The study protocol was approved by the medical ethics committees of both partner sites and registered with the German registry for clinical trials (NR DRKS00023964). This study serves as a first step to investigate FCC practices in a pediatric cardiology setting, providing insight into the relationship between FCC and parent and infant outcomes in a population of infants with CHD. Results will be disseminated in peer-reviewed journals.

## Introduction

Congenital Heart Disease (CHD) is defined as a structural anomaly of the heart or intrathoracic vessels present at birth (1). Incidence of CHD is reported at 9.41 per 1,000 live births worldwide (2). Advances in cardiovascular medicine and surgery have led to improved survival rates of patients with CHD in the past decennia, with an estimated 34.5% decline in mortality between 1990 and 2017 worldwide and an at least 60% decline in Western Europe (3). The majority of children born with CHD now reach adulthood, with a 10-year survival rate of over 80% (4). With this changing demographic, new challenges arise. Patients are confronted with long-term physical health problems, like arrhythmias or heart failure (5). Furthermore, neurodevelopmental delays are frequently observed throughout childhood, with implications for academic achievement, job and life satisfaction (6–8). The cause of these neurodevelopmental delays in this population is hypothesized to be multifactorial and characterized by complex pathways over time (9). Brain dysmaturation, frequently observed in infants with CHD, is suggested as a risk factor, making them neurologically vulnerable to a myriad of stressors early in life, particularly during hospitalization due to open-heart surgery (10). A recent review on the psychosocial development of infants with CHD shows that they are also at risk for emotional, behavioral, and social difficulties (11). The authors hypothesize that hospitalization may overwhelm an infant's capacity to regulate stress, causing these difficulties. Hospitalization is associated with various stressors, for instance invasive procedures, limited mobility, sensory overstimulation, a disrupted parent-infant relationship, or neurotoxic medication. The hospital environment may therefore be an important target for interventions to improve the developmental and psychosocial outcome in this population. A compelling approach to advance the infant's healthcare ecosystem to be more developmentally supportive is Family-Centered Care (FCC), which may promote infant health irrespective of the medical condition (12, 13).

FCC is an international standard of holistic care provision and defined as a health care approach that is respectful of and responsive to the needs and values of a family (14). According to the Institute for Patient- and Family-Centered Care, it comprises four main concepts: (I) respect and dignity, (II) information sharing, (III) participation in care and decision- making and (IV) collaboration between patients, families and the health-care team (www.ipfcc.org). In pediatric care settings, FCC acknowledges the family as the child's primary source of emotional, social and developmental support, and, importantly, as part of the health-care team (15). Accordingly, FCC is a value-based approach that comprises any specific measures and care practices that fall into line with above-mentioned principles. Concrete examples are rooming-in, educating parents/legal guardians, participation of parents/legal guardians in interdisciplinary team rounds, or their presence during medical procedures. Current guidelines for the neonatal, pediatric, and adult Intensive Care Unit (ICU) specify recommendations in the areas of family presence, family support, communication with family members, consultations with specific professional groups, and operational and environmental issues, with varying degrees of scientific evidence (14). Importantly, the endorsement of FCC by professional associations and political institutions is lacking in Europe. The European Foundation for the Care of Newborn Infants has formulated a European Standard of Care for Newborn Health, but it has not been formally implemented in Germany or other European countries. Nevertheless, some of the recommendations may be informally realized in clinical practice. While the evaluation of FCC, by using validated parent satisfaction and experience questionnaires, is implemented as part of a national quality benchmarking tool in the Netherlands, we are not aware of other European countries who routinely evaluate FCC (16). A prospective survey conducted at neonatal intensive care units in Finland, Sweden, Norway, Estonia, Spain and Italy found that FCC practices vary substantially between units with regards to family presence and perceived quality (17).

Most studies on the effects of FCC practices on various physical and mental health outcomes have been conducted with premature infants and their families in neonatal intensive care units, with favorable results. Ding and colleagues recently published a review and meta-analysis of 19 randomized controlled trials investigating the effects of FCC interventions pertaining to educational support, personalized care, parent support, information/communication and environment (18). Results show that FCC interventions improve the clinical outcome of the infant, i.e., weight gain and readmission rates. Above that, these interventions also improve parent-reported outcomes, i.e., satisfaction with care, stress and symptoms of anxiety and depression, which in turn may have an effect on child physical and mental well-being (19).

The holistic nature of the FCC approach aims at minimizing stressors and maximizing a developmentally supporting environment centered on the family-system and falls into line with current insights into child development (20). Accordingly, FCC should be beneficial to various at-risk populations, with potentially lasting effects on both physical health (via immune function and inflammatory processes), and mental well-being (21). Melnyk and colleagues conducted a randomized controlled trial on FCC in a heterogeneous pediatric patient cohort (22). They investigated the effectiveness of an educational-behavioral intervention in mothers of 2- to 7-year old children submitted to a pediatric intensive care unit for various reasons (e.g., respiratory problems, infections, and cardiac problems). The intervention reduced parenting stress levels during hospitalization. Furthermore, similarly to studies in premature babies, the intervention improved maternal mood and reduced symptoms of anxiety and depression. Above that, the intervention had an effect on child mental well-being, specifically externalizing symptoms- a result that warrants further research (22). The results of this study supports the notion that the beneficial effects of FCC may extend to various medical cohorts. In pediatric cardiology, several authors argue that FCC practices may be applicable to infants with CHD, pointing to a mismatch between the neurobiological needs of these infants and the hospital environment (23–29). The neurological vulnerability observed in infants with CHD resembles that of premature infants, most notably with regard to white matter immaturity (30). Two studies investigating in-hospital caregiving interventions for parents of premature infants found positive effects of the intervention on frontal EEG power and cerebral white matter maturation and connectivity of the infant, supporting the notion that a positive early environment stimulates neurodevelopment in this vulnerable group (31, 32). Providing FCC to hospitalized infants with CHD may be similarly beneficial to neurodevelopment and thus improve short-term and long-term outcomes. However, to our knowledge, there is limited empirical support that demonstrates the effectiveness of FCC in this specific population and no studies have been conducted on FCC practices at Pediatric Cardiac Intensive Care Units (PCICUs) caring for children with CHD in Germany.

Primary aim of this cross-sectional two-center study is to investigate FCC practices at two PCICUs in Germany. As infants are limited in their ability to verbalize their needs and experiences during hospital stay, assessing the experiences of parents is crucial for the evaluation of the quality of care (33). We will compare the two participating centers concerning parent satisfaction with FCC and unit adherence to FCC practices in hypothesis generating analyses.

Secondary aim of the study is investigating associations between parent satisfaction with FCC practices and parent, as well as infant outcomes. On the parent level, we hypothesize that higher parent satisfaction with FCC is associated with lower levels of parental anxiety, depression, stress, and parenting stress, similarly to the findings on FCC interventions in premature babies. Accordingly, we will investigate these associations in hypothesis confirming analyses. On the child level, we will investigate associations between parent satisfaction with FCC and child mental and physical well-being as rated by the parents. The analysis will be hypothesis generating, as there is minimal prior research to guide hypotheses about parent satisfaction with FCC and infant mental well-being, and as associations between parent satisfaction with FCC and infant physical well-being may be specific to the underlying medical condition.

## Methods and Analysis

### Design

This two-center cross-sectional study will be conducted at two PCICUs of two specialized pediatric cardiac centers in Germany (Berlin, Bad Oeynhausen).

### Study and Outcome Measures

#### Socio-Demographic Information

Socio-demographic information of the primary caregiver will be assessed with a purpose-designed questionnaire based on German demographic standards (34). The questionnaire comprises questions about age, migration background, first and second language, educational and professional background, current employment status, legal and partnership status of the primary caregiver, as well as number, age and gender of children.

#### Unit Adherence to FCC Practices

We will use the Gap Analysis Tool provided by the Society of Critical Care Medicine (SCCM) Guidelines for Family-Centered Care in the ICU (35). This tool comprises all 24 recommendations of the guidelines by the SCCM, which are rated to indicate how frequently the ward already implements each recommendation as follows: 0 (nearly always), 1 (usually), 5 (sometimes), 10 (nearly never). For recommendations only applicable to neonatal ICUs, a “not applicable” option is available. Each score is transferred into an item score by multiplying the frequency score by outcome points, which are specified for each recommendation. Outcome points range from 1 to 5 depending on the importance of the particular outcome the recommendation targets. Accordingly, item scores range from 0 to 50.

#### Parent Satisfaction With FCC Practices

We will use the EMpowerment of PArents in The Intensive Care-30 Questionnaire (EMPATHIC-30) (36). It is a self-report instrument consisting of 30 statements and assesses satisfaction with experiences at the ICU. The questionnaire is divided into five domains: information (five items), care and cure (eight items), parental participation (six items), organization (five items) and professional attitude (six items). Responses are given on a six-point scoring scale ranging from 1 (“certainly no”) to 6 (“certainly yes”). A separate box “not applicable” is available for all statements. The questionnaire was translated and culturally adapted for use in German-speaking populations by Nagl-Cupal and Lippoldt (37). Latour and colleagues reported a Cronbach's a of 0.93 on the full scale and Cronbach's a ranging from 0.73 to 0.81 on the domain level (36).

#### Complexity of Disease

##### Classification of CHD Severity

We will classify CHD severity according to three diagnostic categories proposed by Warnes and colleagues (38): complex (e.g., truncus arteriosus/hemitruncus, transposition of the great arteries), moderate (e.g., coarction of the aorta, tetralogy of Fallot), and mild (e.g., isolated small ventricular septal defect without associated lesions, isolated congenital aortic valve disease).

##### Aristotle Comprehensive Complexity Score

We will assess the complexity of cardiac disease using the Aristotle Comprehensive Complexity score (ACC) (39). This pediatric risk score is based on expert opinion and ranges from 1.5 to 25. It consists of three dimensions (complexity, procedure-dependent factors, and procedure-independent factors), each comprising several factors, which in turn comprise several variables (three variables for the complexity dimension, 167 for the procedure-dependent factors, and 81 for the procedure-independent factors). Each variable has a reference value that was created by asking medical professionals to score each variable regarding their contribution to mortality, morbidity and difficulty on a scale from 1 to 5, and by computing the median of the scores. The Aristotle Comprehensive Complexity Score predicted 30-day mortality and length of ICU stay during the 1st postoperative week as well as morbidity after congenital heart surgery in previous studies (40, 41).

#### Infant Physical and Emotional Well-Being

We will use the German Version of the Pediatric Quality of Life Inventory [PEDSQL, (42)] to assess both infant physical and emotional well-being as rated by the parents. The acute version for infants aged 1–12 months consists of 36 items and assesses infant well-being within the past seven days. It comprises five subscales (physical functioning, physical symptoms, emotional functioning, social functioning and cognitive functioning). For the assessment of parent-rated physical well-being, we will use the physical health summary score, which consists of the subscales physical functioning (six items) and physical symptoms (10 items). For parent-rated mental well-being, we will use the emotional functioning subscale (12 items). Responses are given on a five-point scale ranging from 0 (“never”) to 4 (“almost always”). The PEDSQL for infants has good internal consistency reliability, with a Cronbach's a of 0.92 for the total scale scores and Cronbach's a of 0.82 and 0.87 for the physical health summary score and the emotional functioning subscale, respectively (42).

#### Parent Emotional Well-Being

We will use the German version of the Depression Anxiety Stress Scale (DASS-21) in order to measure symptoms of depression, anxiety and stress of the parents (43, 44). It is a self-report instrument consisting of 21 items and assesses negative emotional states within the past 7 days. It comprises three subscales with seven items each: depression, anxiety and stress. Responses are given on a four-point scoring scale ranging from 0 (“not at all”) to 3 (“most of the time”). The scale has good internal consistency reliability, with Cronbach's a of 0.90, 0.81, and 0.86, for the subscales depression, anxiety and stress, respectively (45).

#### Parenting Stress

We will assess parenting stress by using the German version of the Parental Stressor Scale: Neonatal Intensive Care Unit [PSS:NICU, (46)]. The German version of the scale consists of 13 items and assesses the extent of stress caused by experiences in the ICU. It comprises two subscales: infant behavior and appearance, and parental role alterations. The third subscale was removed due to a low number of items and unclear factor structure in the German psychometric study. Responses are given on a five-point scale ranging from 1 (“not at all stressful”) to 5 (“extremely stressful”). The scale has good internal consistency reliability, with Cronbach's a of 0.85 for the full scale and Cronbach's a of 0.82 and 0.87 for the subscales infant behavior and appearance, and parental role alterations, respectively (47).

### Participants

Participants will be infants aged 0–12 months, hospitalized for treatment of CHD at one of the two pediatric cardiac centers, and their primary caregiver. The attending physician will recruit participating families during their hospital stay at the participating PCICU. Participation is voluntary.

#### Inclusion Criteria

- Infant is diagnosed with congenital heart disease
- Infant is aged 0–12 months at recruitment
- Hospitalization at the PCICU for ≥12 h
- Primary caregiver has German language proficiency.

#### Exclusion Criteria

- Unclear legal custody status of both parents
- Infant has end-of-life care pathway
- Infant died before recruitment.

### Procedure

At commencement of data collection, the senior physician and senior nurse of each participating PCICU will fill out the Gap Analysis Tool. All parents of infants hospitalized with CHD will be recruited after discharge from the PCICU, upon admission to the normal/intermediary care unit. The attending physician of the intermediary/normal care unit will check inclusion and exclusion criteria and provide oral and written study information to eligible parents. If parents decide to participate, the attending physician will obtain written informed consent from all parents with custody rights. The attending physician will then assess primary caregiver status by asking who took over most caregiving responsibilities (e.g., feeding, bathing, changing diapers) for the child during the past month. The primary caregiver will then receive the questionnaires and a return envelope. They will be asked to complete the questionnaires within 24 h after discharge from PCICU and hand it to one of the staff members of the unit in the sealed envelope, to ensure confidentiality. One of the investigators at each site will collect the returned questionnaires and hand them to a member of the research team not involved in the clinical care for the patients, for data entry. The principal investigator at each site will document the patient characteristics (infant age, gender) and medical information (e.g., diagnoses, duration of stay at PCICU, room size at PCICU) for each participating infant.

### Data Handling

We will use study participant numbers on all documentation to ensure confidentiality. All electronic study-related information will be stored on hospital servers in folders to which only members of the research team have access. One password-protected file linking study participant number and patient identification will be stored separately at each institution. Study information on paper will be kept in locked cabinets with restricted access. Data will be archived for 15 years after completion of data collection. We will conduct data entry in duplicate, in order to check for data entry mistakes. Principal and co- investigators will have access to final data files. Authorship of study reports will be assigned according to contribution to design, conduction, data analysis, interpretation and reporting of the results in writing and oral presentation. Study participants and funding institutions will be informed of the results at the end of the study period.

### Statistical Analyses

#### Power Analysis and Sample Size

Given the annual number of infants with congenital heart disease at the PCICUs of both centers, an inclusion of *N* = 2 × 140 families (140 families per center) is the maximum achievable number in the data collection period of 1 year. A power analysis was performed using G^*^Power for F-test for multivariate ANOVAs and in R for multilevel modeling using the R package “powerlmm.” Primary analyses are hypothesis generating. Secondary analyses are hypothesis confirming for variables on the parent level and hypothesis generating for variables on the child level. Effect sizes for our patient and age group could not be inferred from earlier studies or meta-analyses.

With an alpha level of 5%, a power of 0.80 and an upper bound total sample size of *N* = 2 × 140, the minimum effect size required for statistical detection in an ANOVA is Cohen's *d* = 0.34. This is considered to be a small-to-medium effect size. That same effect size would lead to an estimated power of 74% in a multilevel model with an alpha of 5%, and a total sample size of *n* = 2 × 140, assuming an intraclass correlation of 0.40, and 5,000 simulation runs. The minimum detectable multilevel effect size with a power of 0.80 is Cohen's *d* = 0.37.

#### Analysis Plan

Analysis will be performed in SPSS version 25/27 and R 4.0.2 with the packages “psych,” “mice”and “lmer.”

##### Preliminary Analyses

Given the expected sample size of 140 families for each PCICU, the Central Limit Theorem applies, rendering the assumption of normality in the predictor and/or outcome variables redundant. We will assess linearity of the association between parental FCC satisfaction and FCC adherence. Furthermore, we will assess equality of variances between centers on FCC satisfaction and FCC adherence. Missing data, if any, are evaluated for their origin, being Missing Completely At Random (MCAR), or either Missing At Random (MAR) or Not Missing At Random (NMAR) using Little's MCAR test to probe for the risk of bias in the main analyses (48). If missing values are missing completely at random, missing values are handled within the multilevel models through restricted maximum likelihood estimation. If data are MAR/NMAR, data will be multiply imputed before the main analyses (49). Results for all following analyses will be pooled over imputed datasets. Furthermore, we will report descriptive statistics of and analyze differences between centers concerning socio-demographic characteristics of the primary caregiver and infant medical characteristics.

##### Primary Analyses

In order to investigate differences between the two PCICUs concerning unit adherence to FCC and parent satisfaction with FCC, we will conduct two analyses of covariance (ANCOVA), accounting for relevant individual differences in socio-demographic and medical information as found from the descriptive analyses. First, we will conduct an ANCOVA with EMPATHIC-30 scores as the dependent variable, unit (Berlin, Bad Oeynhausen) as between-subjects factor, and scores on the Gap Analysis Tool as a covariate. Second, we will perform an ANCOVA of GAP Analysis Tool scores between units, with the EMPATHIC-30 scores as a covariate.

##### Secondary Analyses

In order to investigate associations between parent satisfaction with FCC practices and parent, as well as infant outcomes, we will use multilevel models with PCICU as the level two factor. Such models account for shared exposure to unit characteristics, causing families within a unit to be more similar (to an unknown extent) compared to families from different units.

For the analysis of outcomes on the parent level, we will enter parent satisfaction with FCC as independent variable. Measures of parent well-being (specifically, symptoms of anxiety, depression, stress and parenting stress) will be entered as dependent variables. Extended models will be built to account for potential effects of infant characteristics (e.g., age and gender), medical variables (e.g., duration of stay at hospital, complexity of disease), as well as socio-demographic variables (e.g., employment status, ethnicity or professional background).

For each measure of parent well-being (symptoms of anxiety, depression, stress and parenting stress), a series of multilevel models is estimated. Each model in the set has incremental complexity. All models will be estimated using Restricted Maximum Likelihood, defining the covariance structure through variance components, and using the Huber- White sandwich estimator for the standard errors (50, 51).

For *each* parent well-being measure separately, the series of hypothesis confirming models is outlined below:

1) Model 1 estimates the unconditional means model for the parent well-being score.
2) Model 2 estimates the conditional means model, adjusting for infant characteristics, medical and socio-demographic variables.
3) Model 3 estimates the random intercept for the fixed slope association between FCC satisfaction and parent well-being, adjusted for infant characteristics, medical and socio-demographic characteristics.
4) Model 4 estimates the random intercept and random slope for the association between FCC satisfaction and parent well-being, adjusted for infant characteristics, medical and socio-demographic characteristics.

For the analysis of outcomes on the child level, we will enter parent satisfaction with FCC as independent variable. Measures of infant well-being (specifically, mental and physical well-being as rated by the parents) will be entered as dependent variables. Extended models will explore potential effects of infant characteristics (e.g., age and gender), medical variables (e.g., duration of stay at hospital, complexity of disease), as well as socio-demographic variables (e.g., employment status, ethnicity or professional background). Two series of models are estimated for child well-being (parent-rated physical and parent-rated mental well-being). Similar to above analysis, each model in the set has incremental complexity and all models will be estimated using Restricted Maximum Likelihood, defining the covariance structure through variance components, and using the Huber-White sandwich estimator for the standard errors.

For each child well-being measure separately, the series of hypothesis generating models is outlined below:

1) Model 1 estimates the unconditional means model for the child well-being
2) Model 2 estimates the conditional means model, adjusting for infant characteristics, medical and socio-demographic variables.
3) Model 3 estimates the random intercept for the fixed slope association between FCC satisfaction and child well-being, mutually adjusted and adjusted for infant characteristics, medical and socio-demographic variables.
4) Model 4 estimates the random intercept and random slope for the association between FCC satisfaction and child well-being, adjusted for infant characteristics, medical and socio-demographic variables.

In all series of models, the change in model deviance between subsequently nested models is evaluated at a 5% significance level using the Likelihood Ratio Test.

## Discussion

Family-centered care is a compelling framework for the care for children with physical illness. Both premature infants and children with CHD have a risk for brain immaturity and are therefore neurologically vulnerable for stressors early in life. A protective environment during hospitalization may improve the mental and physical well-being of those infants. Our primary aim is to inventory FCC practices at two PCICUs in Germany. Results will provide insight into current FCC practices, the parents' experiences of these practices, and potential changes that can be implemented at those centers to further improve FCC practices. Results may be applicable to other centers in Germany and Europe. Secondary aim of the study is investigating associations between parent satisfaction with FCC practices and mental well-being of the parents, as well as physical and mental well-being of the child. These results will inform us about associations between parent experiences with FCC and parent and infant outcomes in pediatric cardiology. They may provide the basis for international follow-up studies in this population, with more elaborate, longitudinal and experimental research designs, in order to advance insights into the benefits of FCC in this population.

As this study is conceptualized as cross-sectional, this study is time efficient. Filling out the questionnaires will take ~30 min in total. Implementation of the study will be closely monitored at both centers and time expenditure for medical stuff recruiting the families at the normal/intermediary ward is manageable. We therefore expect a high participation rate. Results will be representative for the population of primary caregivers with hospitalized infants with CHD. Due to the cross-sectional study design, no cause-and-effect relationship can be established between any of the variables. As part of the research questions are hypothesis generating, results will have to be replicated in hypothesis confirming research. Randomized clinical follow-up trials will be essential. Additionally, follow-up studies should ideally include qualitative research methodologies, such as interviews or focus groups, to provide rich data for an in-depth exploration of the topic (52).

Family-centered care potentially provides effective secondary prevention to medically vulnerable cohorts, particularly to children who are at risk for neurodevelopmental sequelae. Above that, the framework facilitates a value-based discourse about modern health care, putting theoretical paradigms like post-colonialism, critical feminism, relational inquiry and intersectionality on the agenda (53). It may therefore serve as counterbalance in increasingly economized systems, which exerts pressure on health care providers (54–57). Studying FCC in infants with CHD is an important step to optimize care for this population.

## Ethics Statement

The study was approved by the Ethics Committee at Charité Berlin (EA2/032/20) and the Ethics Committee at Ruhr-University Bochum (2020-713). Written informed consent to participate in this study will be provided by the participants' parents with custody rights. The study was registered in the German registry for clinical trials (Deutsches Register Klinischer Studien, www.drks.de) with registration number DRKS00023964.

## Author Contributions

HF, KS, RR, and JL conceptualized the study. HF wrote the first draft of the manuscript. RR contributed the analysis plan. All the authors critically reviewed and contributed to the final draft of the manuscript.

## Conflict of Interest

The authors declare that the research was conducted in the absence of any commercial or financial relationships that could be construed as a potential conflict of interest.

## Publisher's Note

All claims expressed in this article are solely those of the authors and do not necessarily represent those of their affiliated organizations, or those of the publisher, the editors and the reviewers. Any product that may be evaluated in this article, or claim that may be made by its manufacturer, is not guaranteed or endorsed by the publisher.
